# Non-Crosslinked Hyaluronic Acid Redensity 1^®^ Supports Cell Viability, Proliferation, and Collagen Deposition in Early Burn Management

**DOI:** 10.3390/pharmaceutics18010021

**Published:** 2025-12-23

**Authors:** Zhifeng Liao, Xi Chen, Romain Brusini, Jimmy Faivre, Lee Ann Applegate, Killian Flegeau, Nathalie Hirt-Burri

**Affiliations:** 1 Faculty of Biology and Medicine, University of Lausanne, CH-1005 Lausanne, Switzerland; liao.zhifeng@unil.ch (Z.L.); xi.chen.1@unil.ch (X.C.);; 2 Service of Plastic, Reconstructive, Esthetic & Hand Surgery, Lausanne University Hospital, CH-1011 Lausanne, Switzerland; 3 Research and Development Department, Teoxane SA, Rue de Lyon 105, CH-1203 Geneva, Switzerland; r.brusini@teoxane.com (R.B.); j.faivre@teoxane.com (J.F.); 4 Center for Applied Biotechnology and Molecular Medicine, University of Zurich, CH-8057 Zurich, Switzerland; 5 Oxford OSCAR Suzhou Center, Oxford University, Suzhou 215123, China

**Keywords:** biomaterials, burn wound healing, hyaluronic acid hydrogels, primary cells, regenerative medicine, scarring, stem cell delivery, tissue engineering, wound healing

## Abstract

**Background/Objectives:** Burn injuries pose a significant challenge due to tissue damage and impaired healing. Cell-based therapies offer promise by delivering therapeutic cells to the wound site. However, effective cell delivery remains a critical hurdle. This study investigates the potential of non-crosslinked hyaluronic acid (HA) as a simple, versatile carrier for delivering autologous keratinocytes and fibroblasts to treat early burn wounds. **Methods:** Primary keratinocytes and fibroblasts were isolated from uninjured adult skin. In addition, fibroblasts and adipose stem cells (ASC) from polydactyly and progenitor fibroblasts were used. Non-cross-linked HA Redensity 1^®^ (RD1) solutions of varying concentrations were prepared and applied to various in vitro models. Cell viability, proliferation, migration, and collagen stimulation were assessed using standard assays. Additionally, cells were suspended in Redensity 1 and applied to an in vitro de-epidemalized dermis (DED) wound model to examine cell delivery and tissue reformation. **Results:** Preliminary data demonstrated the feasibility of using non-cross-linked HA RD1 gel as a cell carrier. RD1 gel enhanced cell viability, retention, migration, and collagen deposition. Histological analysis revealed improved cell adhesion and migration. **Conclusions:** This study provides valuable insight into the potential of non-cross-linked HA RD1 as a simple and effective delivery vehicle for cell therapies in early burn care. Successful translation of this approach could significantly improve clinical outcomes for burn patients.

## 1. Introduction

Burn injuries pose a significant global health burden, resulting in substantial morbidity and mortality [1,2,3,4,5]. While acute management focuses on preventing infection and fluid resuscitation, the subsequent healing process often presents challenges. Extensive burn wounds can lead to significant scarring, contractures, and impaired functional recovery, necessitating prolonged rehabilitation and impacting the patient’s quality of life [6,7,8].

Traditional wound healing approaches often rely on autografts, which involve harvesting skin from uninjured areas of the patient. However, autografts can be limited by donor site morbidity, inadequate graft availability, and potential complications [9,10,11,12]. Furthermore, the use of allografts, derived from deceased donors, carries the risk of disease transmission and immune rejection [13,14].

In recent years, regenerative medicine has emerged as a promising avenue for burn wound treatment, aiming to accelerate and enhance the healing process. Cell-based therapies have garnered significant attention due to their inherent regenerative potential [15,16,17,18,19,20,21]. Different cell types, such as fibroblasts, keratinocytes, epidermal stem cells, multipotent mesenchymal stromal cells, bone marrow stem cells, adipose-derived stem cells, and induced pluripotent stem cells, were used to promote wound healing [22,23,24,25,26,27,28], and these cell sources were reviewed by Rangatchew in 2021 [7].

However, direct application of cells at the wound site often results in limited cell retention and engraftment due to factors including cell survival, rapid cell migration, lymphatic drainage, and immune rejection [7,10,11]. Therefore, an ideal scaffold that has the capacity to act as a template for cell therapy is essential [29,30,31]. Several key factors must be considered when using a scaffold as the delivery system, which include cytocompatibility, biocompatibility, biodegradability, mechanical strength, and ideal internal and external architecture to support cell migration and nutrient diffusion.

Biomaterials are essential components in various burn dressings and tissue-engineered constructs. Their primary purpose is to replicate the skin’s extracellular matrix (ECM), which comprises collagen, elastin, proteoglycans, nidogen, laminin, and perlecan. The main key components confer critical properties: collagen provides structural strength, elastin enables elasticity and flexibility, and proteoglycans contribute hydration and viscosity [31].

To date, the majority of commercially available products for skin burn and wound management are based on collagen, decellularized tissues, or hydrogel [28,32,33]. Among these biomaterials, hydrogels have proven particularly favourable for burn healing by providing an optimal microenvironment and optimal hydration, and are extensively utilized in tissue engineering approaches [34,35]. These three-dimensional networks composed of hydrophilic polymers exhibit excellent biocompatibility, tunable mechanical properties, and the ability to mimic the ECM of native tissues. By incorporating cells within a hydrogel scaffold, researchers can create a more physiologically relevant environment that supports cell survival, proliferation, and function, along with providing an ideal methodology for large surface treatments [36]. Among the various types of hydrogels explored for biomedical applications, hyaluronic acid (HA)-based gels (overall structure provided in Supplementary Figure S1) have gained particular attention due to HA’s intrinsic biocompatibility, biodegradability, and role in tissue regeneration. HA or hyaluronan is a key component in a variety of medical, pharmaceutical, nutritional, and cosmetic applications. HA is used in medical applications comprising drug delivery, cancer therapy, wound treatment, orthopedics, ophthalmology, arthrology, pneumology, urology, otolaryngology, odontology, or cosmetic and dietary applications [34].

The burn wound healing product based on hyaluronic acid should be a commercially available medical device that meets the CE or FDA standards and should possess all the necessary criteria, such as quality, efficacy, and safety. However, few hyaluronic acid-based scaffold products were used for wound healing processes [37], which limits the clinical development of such approaches.

In this study, we tested Redensity 1^®^ (RD1), a CE-MDR-approved, class III, injectable medical device, as a biocompatible vehicle for cell delivery. RD1 is formulated with 1.5% *w*/*w* of high-quality, high molecular weight, non-animal origin hyaluronic acid, 0.3% *w*/*w* lidocaine for patient comfort and pain alleviation, and a cocktail of nutrients (including amino acids, minerals, antioxidants, and vitamins). RD1 is indicated for the enhancement of skin quality, hydration, and correction of withered skin marked by signs of ageing on the face, neck, and neckline. It is also indicated for the prevention of superficial fine wrinkles, including forehead lines, crow’s feet, perioral rhytids, and necklines. As a light, fluid, and smooth product, RD1 is typically injected superficially into the dermis or subdermal plane for optimal effectiveness and would represent an ideal candidate for cytotherapies in burn wound healing [38,39].

## 2. Materials and Methods

### 2.1. Ethical Compliance of the Study

Obtention and use of patient primary cellular materials (i.e., primary polydactyly fibroblasts and ASCs, adult keratinocytes) followed the regulations of the Biobank of the Department of Musculoskeletal Medicine at the CHUV (Lausanne University Hospital, Lausanne, Switzerland) and were registered in the CHUV-DAL Department Biobank under an ethics protocol approval and following the applicable biobanking directive (BB_029_DAL). The clinical-grade primary progenitor cell source used in the present study (i.e., FE002-SK2 primary progenitor fibroblasts) was established from the FE002 organ donation, as approved by the Vaud Cantonal Ethics Committee (University Hospital of Lausanne, Ethics Committee Protocol N 62/07).

### 2.2. Cell Sources

Polydactyly primary dermal fibroblasts and adipose-derived stem cells (ASCs) from 3 polydactyly patients (aged from 1, 2, and 7 days) were thawed from previously established cell banks [40]. Dermal fibroblasts were cultured in Medium 1 composed of Dulbecco’s Modified Eagle Medium (DMEM, Gibco, Thermo Fisher Scientific Waltham, MA, USA) supplemented with 10% fetal bovine serum (FBS, Merck, Darmstadt, Germany), 1% L-glutamine (Gibco, Thermo Fisher Scientific), and 1 mM sodium pyruvate (Gibco, Thermo Fisher Scientific). ASCs were cultured in Medium 2, composed of DMEM supplemented with 5% human platelet lysate (HPL, Sexton Biotechnologies, Indianapolis, IN, USA). HaCaT immortalized keratinocytes (ATCC, Manassas, VA, USA) were cultured in Med_1, served as a control cell line. Primary adult keratinocytes were obtained from tissue designated as medical waste following routine abdominoplasties as described previously [8] and cultured in Medium 3, CnT-PR medium (CellnTec, Bern, Switzerland). FE002-SK2 primary progenitor fibroblast vials from our clinical cell bank were thawed and serially expanded in vitro in Med_1. For all the cells, medium was exchanged twice per week, and all cultures were maintained in humidified incubators at 37 ◦C with 5% CO_2._ Primary cells were used for experiments between passages 3 and 6 unless specified, and FE002-SK2 primary progenitor fibroblasts were used at passages 7 and 9.

### 2.3. RD1 Cytotoxicity Assay on FE002-SK2 and Polydactyly Fibroblasts and ASC Cells

RD1 is a 15 mg/g non-crosslinked hyaluronic acid solution with a viscosity of 2 Pa.s (1 Hz, 25 °C). Unlike crosslinked dermal fillers that persist for months to years, uncrosslinked (native) HA exhibits a half-life of less than 24 h in the skin due to rapid enzymatic degradation by hyaluronidases [41]. This short residence time makes RD1 suitable as a cell carrier, providing initial hydration without hindering tissue regeneration. RD1 is a CE-marked Class III medical device that has undergone all required biocompatibility testing in accordance with the ISO 10993 series [42], demonstrating its safety for clinical use. Furthermore, RD1 has been commercially available for several years without any reported adverse events.

FE002-SK2 primary progenitor fibroblasts, Polydactyly fibroblasts, and ASCs were seeded at a cellular density of 1 × 10^4^ cells/well in 96-well plates. After 24 h, cells were treated with 100 µL RD1 gel diluted in their respective culture medium at concentrations ranging from 100% to 3.125% RD1 gel. Cells cultured in their respective media served as a positive control, and cells maintained in PBS served as a negative control. Cell viability was assessed after 72 h using the CellTiter^®^ assay (Promega, Madison, WI, USA) as recommended by the manufacturer.

### 2.4. Cellular Growth Evaluation of Various Cell Types Exposed to Hydrogels

We developed an in vitro model to mimic gel diffusion in the skin with the use of 100 µL cell strainer inserts (CSI) (Corning^®^, Corning, NY, USA). Polydactyly fibroblasts and ASCs cells were seeded at 3 × 10^3^ cells/cm^2^ in 6-well plates in their respective culture medium. After 24 h, 3 culture conditions were set up: (1) control, cells in their respective culture medium with no CSI (CTRL), (2) CSI alone, cells in their respective culture medium with a CSI and no RD1 gel (3) CSI with 100 µL RD1, cells in their respective culture medium with a CSI containing 100 uL of RD1 gel (see Supplementary Figure S2 for the experimental set up). In brief, the medium was removed from the cultured cells and replaced with 3 mL of fresh medium. Cell strainers were placed in the 6-well plates with and without 100 µL RD1 gel. Then, medium was added to submerge the gel (final volume of 9 mL). There were no further medium changes during the experiment duration. Cells were enumerated after 4 and 7 days. Results were expressed as a percentage of the control. Pictures were taken with an inverted Olympus IX83 microscope (Olympus, Tokyo, Japan) with a DP75 camera (Olympus).

### 2.5. Collagen Production of Various Cell Types

To evaluate the collagen production enhancement ability of RD1, polydactyly fibroblasts and FE002-SK2 skin progenitor fibroblasts were seeded at 3 × 10^3^ cells/cm^2^ in 6-well plates. Upon reaching confluence, 3 groups and controls were set up as follows: (1) control, cells in culture medium 1 (CTRL), (2) CSI with 100 µL RD1, cells in culture medium 1 with a CSI containing 100 uL of RD1 gel, (3) VitC, cells in culture medium 1 supplemented with 10^−4^ M vitamin C. The medium was replaced every 2 days for 7 days for the control and the vitamin C condition, but not for the CSI with the RD1 gel. After 7 days, the treated cell monolayers were washed with 1× PBS, fixed in methanol at −20 °C for 10 min, rinsed three times with 1× PBS, and Sirius Red staining and subsequent quantification were performed. The collagen was stained with 2 mL of 0.1% Sirius Red (Abcam, Cambridge, UK) in 1.3% picric acid (Merck, Darmstadt, Germany) for 1 h at room temperature with agitation; after gently rinsing each well four times with 0.5% acetic acid. Macroscopic imaging was performed on an iPhone 12 Pro Max (Apple, Cupertino, CA, USA) and microscopic images on an Olympus IX83 microscope with a DP75 camera. For quantification, 2 mL of 0.1 M NaOH was added to the wells for 30 min at room temperature with agitation. The samples were then transferred to a new 96-well plate for absorbance measurement at 560 nm on a Varioskan LUX multimode plate reader (Thermo Fisher Scientific, Waltham, MA, USA). Absorbance data were analyzed using the Skanit-RE software v5.0 (Thermo Fisher Scientific, Waltham, MA, USA).

### 2.6. Adipogenesis Induction Assays with Polydactyly Adipocyte Stem Cells (ASC)

To assess whether RD1 gel was able to induce the differentiation of polydactyly ASC cells in natural conditions, cells were seeded at 3 × 10^3^ cells/cm^2^ in 6-well plates. Upon reaching confluence, 3 groups and controls were set up as follows: (1) positive control for ASC differentiation with adipogenic induction medium (DMEM, ITS 1× (Corning^®^, NY, USA), 100 μM indomethacin (Acros Organics™, Thermo Fisher Scientific, USA), 1 μM dexamethasone (Acros Organics™, Thermo Fisher Scientific, USA), 100 μM IBMX (Alfa Aesar™, Thermo Fisher Scientific, USA), (2) negative control for ASC differentiation with culture medium 2, and (3) CSI with 100 µL RD1 in culture medium 2. Medium was changed once a week for the RD1 group and twice a week for the other 2 groups. After 14 days of adipogenic induction, lipid droplet formation was assessed by Oil Red O staining (Sigma-Aldrich^®^, USA). Experiments were performed on 3 different polydactyly ASC sources.

### 2.7. Analysis of Adipogenic Gene Induction (PPARγ, LPL): RNA Extraction and Quantitative Real-Time PCR

Total RNA was extracted using the TRIzol reagent (Thermo Fisher Scientific, Waltham, MA, USA) as described by the manufacturer. RNA purity and concentration were quantified via spectrophotometry with a NanoDrop instrument (Thermo Fisher Scientific, Waltham, MA, USA). Reverse transcription into cDNA was performed using 500 ng of RNA in a final volume of 10 µL using a PrimeScript RT reagent kit (Takara Bio, San Jose, CA, USA) according to the manufacturer’s protocol. The reverse transcription cycle conditions were as follows: 37 °C for 15 min and 85 °C for 5 s. A real-time polymerase chain reaction (RT-PCR) was then performed in 96-well microplates on a QuantStudio™ PCR system (Thermo Fisher Scientific, Waltham, MA, USA). The reaction was performed using 5 ng of cDNA for a final volume of 10 µL using the KAPA SYBR Fast (Roche, Basel, Switzerland) according to the manufacturer’s protocol. Fluorescence was acquired using the following cycling conditions: 95 °C for 3 min (enzyme activation) and 40 amplification cycles (95 °C for 3 s and annealing extension at 60 °C for 30 s). Each sample was run in triplicate, and the relative expression level for each gene was normalized to GAPDH forward 5′-CGC TCT CTG CTC CTC CTG TT-3′, reverse 5′-CCA TGG TGT CTG AGC GAT GT-3′. Gene expression levels for PPARγ forward 5′-TAC TGT CGG TTT CAG AAA TGC C-3′, reverse 5′-GTC AGC GGA CTC TGG ATT CAG-3′, and LPL forward 5′-CTG GTC AGA CTG GTG GAG CA-3′, reverse 5′-ACA AAT ACC GCA GGT GCC TT-3′ genes were quantified using the 2^−∆∆Ct^ calculation method [43].

### 2.8. Cellular Behavior in Gels

To analyze the possibility of using RD1 gel as a delivery system for cellular therapies, the viability of polydactyly fibroblasts and ASC from 3 donors, as well as the clinical grade FE002-SK2 primary progenitor fibroblast cell line, primary keratinocytes, and HaCaT cells mixed with the gel, was evaluated at different time points. To this end, suspensions containing 250,000 cells were centrifuged at 230× *g* for 10 min, and the supernatant was removed. Cells were mixed with 250 µL of RD1 (1000 cells/µL), and 100 µL was deposited on the bottom of a 96-well plate. The plate was placed in a humidified incubator at 37 °C under 5% CO_2_ for 24, 48, 72 h, and 1 week, at which time live/dead (Biotium, Fremont, CA, USA) staining was conducted. 100 µL of live/dead solution was deposited on the top gel and incubated for 30 min in the incubator at 37 °C under 5% CO_2_. After the incubation period, 20 µL of the mix of cells in RD1 was transferred to the top of a glass slide, and pictures were taken with a fluorescence microscope (Olympus IX83 microscope with a DP75 camera) using the appropriate filters, GFP: green fluorescence for live cells, and TRITC, red fluorescence for dead cells. Cell counting (live and dead cells) was performed with the ImageJ program [44]. Three experiments were conducted for each cell source. Results are presented as a percentage of live cells. Supplementary Figure S3 shows a representative image of the results obtained after live/dead staining.

With the same protocol, cellular survival was evaluated in different storage conditions, such as 4 °C (in the fridge), room temperature (RT) on the bench (with natural light exposure), RT in the dark, and 37 °C (cell culture incubator) for 24 and 48 h.

### 2.9. Cell Delivery Assessment in a (De-Epidermialized Dermis) DED Model

To assess the potential of cellular delivery of cells mixed with RD1 gel, we used a de-epidermialized dermis (DED) model as described previously [8,45]. In brief, DED 1.5 cm^2^ were taken from stored batches at 4 °C, washed several times with DMEM, and incubated for at least 1 h in Medium 1. Then, DED samples were deposited (reticular dermis facing down) onto a perforated metal support measuring 1 cm × 1 cm × 0.5 cm that was placed at the bottom of a 12-well plate. Medium 4 composed by DMEM and Ham’s F12 (Merck, Darmstadt, Germany) at a 3:1 proportion, 0.14 nM cholera toxin (Lubio Science, Zurich, Switzerland), 332.9 ng/mL hydrocortisone (Pfizer, New York, NY, USA), 8.3 ng/mL EGF (Merck, Darmstadt, Germany), 832.2 µM L-glutamine (Thermo Fisher Scientific, Waltham, MA, USA), 0.12 U/mL insulin (Novo Nordisk Pharma, Bagsværd, Danemark), and 10% FBS (Merck, Darmstadt, Germany) was added just to let the top of the dermis uncovered to create an air liquid interface. A glass insert was carefully positioned in the center of the DED with the help of a sterile forceps for the cells suspended in medium, then cells were added on the insert or directly on the DED as follows: (1) 100 µL of RD1 mixed with 20,000 FE002-SK2 fibroblasts (2) 100 µL of RD1 mixed with 20,000 primary keratinocytes (3) 100 µL of RD1 mixed with 20,000 HaCaT cells, (4) 100 µL of RD1 mixed with 4′000 FE002-sk2 and 16′000 primary keratinocytes and (5) 100 µL of culture medium mixed with 20,000 HaCaT cells as the positive control (see Supplementary Figure S4). The negative control was the DED with no cellular addition. The plates with DEDs were incubated for 3 days at 37 °C and 5% CO_2_. Following this period, the inserts were removed, the DED culture medium was refreshed, and the constructs were maintained for 1 week at 37 °C and 5% CO_2_. Then, DED samples were stained with MTT to assess cellular viability and homogeneity.

### 2.10. DED Processing to Evaluate Cellular Attachment and Viability

Cellular attachment and viability were assessed by an MTT staining (3-(4,5-dimethylthiazol-2-yl) 2,5-diphenyltetrazolium bromide conversion assay, Thermo Fisher Scientific, Waltham, MA, USA). The DED were placed into 12-well plates, and 1–2 mL of 0.5 mg/mL MTT in 1 × PBS (Bichsel, Unterseen, Switzerland) was added, followed by a 2-h incubation at 37 °C. After incubation, the samples were rinsed twice with PBS, and imaging was carried out macroscopically using an iPhone 12 Pro Max (Apple, Cupertino, CA, USA). Metabolically active cells appeared stained in purple. The stained DED samples were then fixed in formalin for histological analysis and subsequent hematoxylin and eosin (H&E) staining of 7 µm sections as described previously [45]. Pictures were taken with an inverted Olympus IX83 microscope with a DP75 camera.

### 2.11. Statistical Analysis and Data Presentation

All statistical analyses were performed using GraphPad Prism version 10.4.1 (GraphPad Software, San Diego, CA, USA). For pairwise comparisons, unpaired two-tailed Student’s *t*-tests were applied to assess differences between two independent groups. IC_50_ values were calculated by nonlinear regression using a four-parameter logistic model (variable slope) fitted to dose–response curves.

For experiments involving two independent factors, a two-way analysis of variance (ANOVA) was conducted to evaluate the main effects of each factor and their interaction. When significant effects were detected, post-hoc multiple comparisons were performed using Tukey’s honestly significant difference (HSD) test to identify specific group differences while controlling for family-wise error. Assumptions of normality and homogeneity of variances were verified using Shapiro–Wilk and Levene’s tests, respectively. Statistical significance was set at *p* < 0.05 for all analyses.

## 3. Results

### 3.1. Cytotoxicity Assay for RD1 Gel

**Figure 1:**
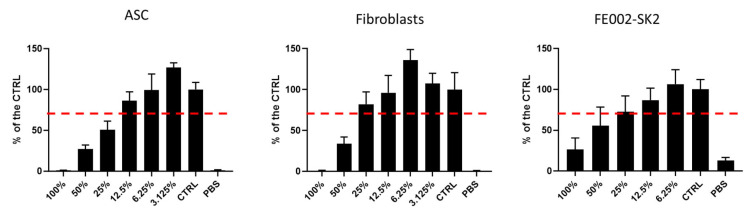
RD1 cytotoxicity assay. CellTiter results of cytotoxicity assay of RD1 gel on ASCs, polydactyly primary fibroblasts, and FE002-SK2 progenitor fibroblast cells. Cellular viability is shown as a % of the control (i.e., untreated cells in growth medium). IC_50_ for ASC cells (25.48%), fibroblasts (45.56%), and FE002-SK2 (60.33%). IC_50_ calculation can be found in Supplementary Figure S5. ASC, adipose stem cells. The dotted red line corresponds to the regulatory threshold at 70% of viability vs. CTRL.

We first evaluated the RD1 gel cytocompatibility with multiple therapeutic cell types, including polydactyly ASC and adult fibroblasts, as well as FE002-SK2 progenitor skin fibroblasts, which are clinical cells used to treat second- and deep-second-degree burns [29,46,47,48,49,50]. As seen in Figure 1, a dose response with an IC50 of 25% was evidenced for ASC cells, 45.56% for fibroblasts, and 60.33% for FE002-SK2 (IC50 calculation described in Supplementary Figure S5). Regarding the 100% gel condition, cells are cultured in undiluted gel without any culture medium, meaning they are completely deprived of essential nutrients for 24 h. Under such extreme conditions, maintaining approximately 60% cell viability is actually quite remarkable and suggests that RD1 may exert a cytoprotective effect rather than a cytotoxic one. In clinical practice, RD1 would be rapidly diluted by interstitial fluids, providing essential nutrients and oxygen for cell survival—a fundamentally different environment from the in vitro 100% gel condition. Visual observations of cell morphology matched the cellTiter assay results (Supplementary Figure S6). In addition, we have also tested a control of the potential cytotoxicity of lidocaine as it was a component of the RD1 gel (Supplementary Figure S7), and we have found that the composition of the RD1 gel with lidocaine shows no statistical difference from that with HA alone. Furthermore, when lidocaine was tested alone, there was a significant decrease in cellular viability.

### 3.2. Clinical Doses of RD1 (100 µL) Induce Polydactyly Fibroblasts and ASC Proliferation

**Figure 2:**
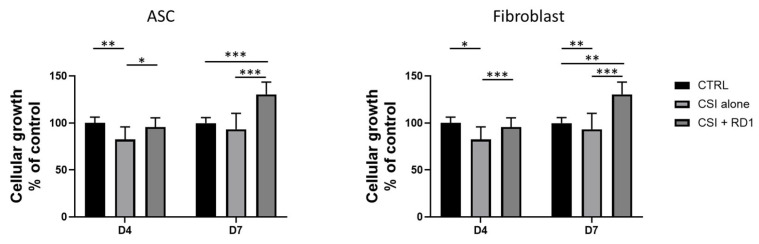
Cellular growth evaluation by cellular enumeration at day 4 and day 7 of polydactyly primary ASCs and fibroblasts cultured in their respective growth medium with the presence of a CSI, with or without 100 µL RD1 gel. Experimental values were normalized to the corresponding control at each time point and expressed as a percentage of control (control = 100%). Data from four independent experiments (n = 4) are presented as mean ± SEM. Statistical comparisons between groups were performed using GraphPad Prism unpaired two-tailed *t* tests. Significant differences are indicated by * *p* < 0.05, ** *p* < 0.01, *** *p* < 0.001.

To capture the effect of clinically relevant doses of RD1 on skin cell proliferation (ASC and polydactyly fibroblasts), an in vitro model using cell strainer inserts (CSI) was developed to mimic RD1 gel diffusion into the skin (Supplementary Figure S2). Figure 2 shows that RD1 promoted cellular growth of both polydactyly fibroblasts and ASC in a significant manner as soon as 4 days of incubation compared to the CSI condition (mean difference = 13.2; 95% CI: 1.5–24.9; *p* = 0.029 for ASC and mean difference = 32.33; 95% CI: 17.05–47.62; *p* = 0.0002 for fibroblasts,) no significant difference was observed at 4 days between the control and the RD1 condition and confirmed at 7 days with a high difference between the control and the RD1 condition (mean difference = 30.52; 95% CI: 20.3–40.6; *p* <0.0001 for ASC and mean difference = 32.33; 95% CI: 11.8–53.4; *p* = 0.036 for fibroblasts). The enumeration data can be found in the Supplementary Figure S8. CSI alone shows a negative impact on cellular growth, which could be due to the physical interaction with cells and suffocation. Therefore, the presence of RD1 significantly improved the cellular growth of both tested primary cells. Images of the cells in culture can be found in the Supplementary Figure S9.

### 3.3. RD1 (100 µL) Induces Collagen Production by Polydactyly Fibroblasts and FE002-SK2 Primary Progenitor Fibroblasts

**Figure 3:**
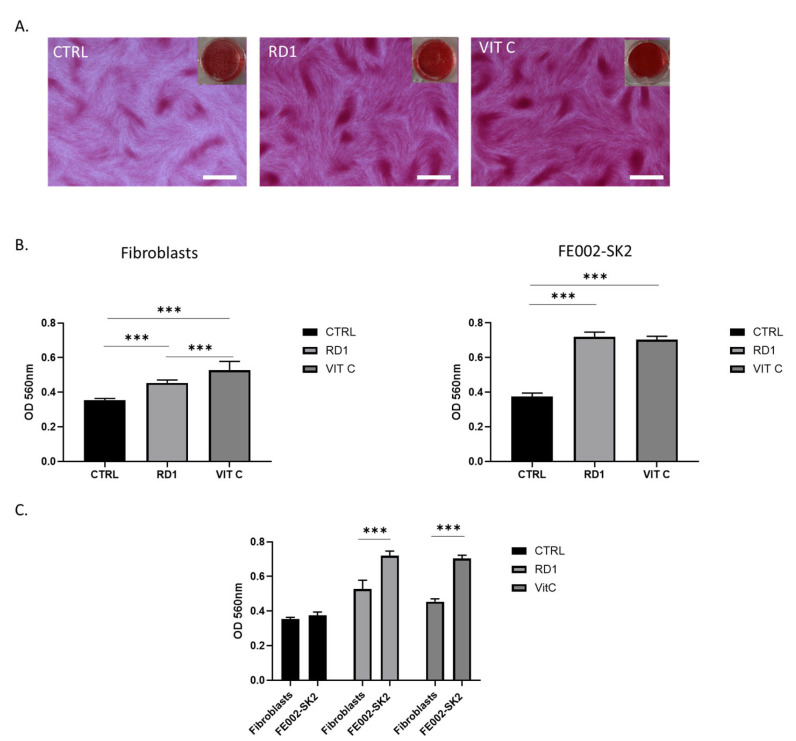
Collagen production: effect of 100 µL of RD1 on fibroblasts. (**A**) Example of Sirius Red staining of polydactyly fibroblasts post-7 days exposure to 100 µL of RD1 or 10^−4^ M Vitamin C. Scale bars = 400 nm (**B**) Collagen quantification by Sirius Red optical density measurements. Raw OD_560_ visible absorbance (which corresponds to collagen content) was measured for three independent experiments. Here are shown the results of one of the experiments. The other two can be found in Supplementary Figures S10 and S11. (**C**) Collagen quantification by Sirius Red optical density measurements. Raw OD_560_ visible absorbance (which corresponds to collagen content) of one experiment where both primary cell lines were treated in parallel. FE002-SK2 shows a higher relative and innate quantity of collagen. Statistical comparisons between groups were performed using GraphPad Prism unpaired two-tailed *t* tests. Significant differences are indicated by *** *p* < 0.001.

Using the same in vitro model with CSI, we assessed the ability of small doses of RD1 gel (100 µL) to promote collagen deposition from polydactily fibroblasts and FE002-SK2 progenitor fibroblast cells. Figure 3 shows that with a dose equivalent to 1.1% of gel (i.e., 100 µL of gel in 9 mL of medium), RD1 gel is able to enhance the collagen production in both polydactyly fibroblasts and FE002-SK2 cells, comparable to the positive control, vitamin C. Figure 3C further demonstrated that FE002-SK2 innately produce significantly more collagen than polydactyly primary cells and thus have already a maximum collagen content confirming previous results [51].

### 3.4. RD1 (100 µL) Does Not Induce ASC Differentiation into Adipocytes

**Figure 4:**
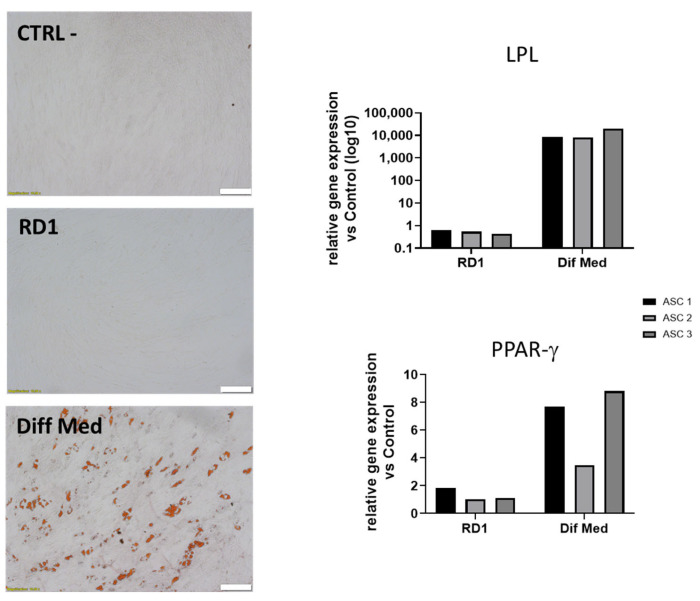
Evaluation of adipogenic potential of 100 µL RD1 on polydactyly ASC primary cells. (**Left**) Representative images of the Oil red O staining for one polydactyly ASC cell line. Scale bar = 100 μm. (**Right**), relative mRNA expression of LPL and PPAR-γ genes. Three polydactyly ASC cell lines were cultured for 14 days in adipocyte differentiation medium (Diff Med), CSI with 100 µL RD1 in culture medium (RD1), and with culture medium (CTRL-). Experiments were performed once for each cell source.

We further assessed the ability of small doses of RD1 gel (100 µL) to induce ASC differentiation into adipocytes in normal culture medium with no addition of differentiation factors. The experiment was performed on three different polydactyly cell sources. Figure 4 shows that RD1 does not influence ASC differentiation into adipocytes in normal conditions, as shown with Oil Red O staining as well as with qPCR, where we observe no increased expression of LPL and PPAR γ genes. Proliferator-activated receptor gamma (PPAR-γ) controls the transcription of numerous genes involved in lipid uptake, storage, and insulin sensitivity, and its upregulation is characteristic of adipocyte commitment. Lipoprotein lipase (LPL) serves as a functional marker of adipogenesis; elevated LPL expression reflects the acquisition of lipid-handling capacity characteristic of differentiated adipocytes.

### 3.5. RD1 Maintains Cell Viability in Starving Conditions

**Figure 5:**
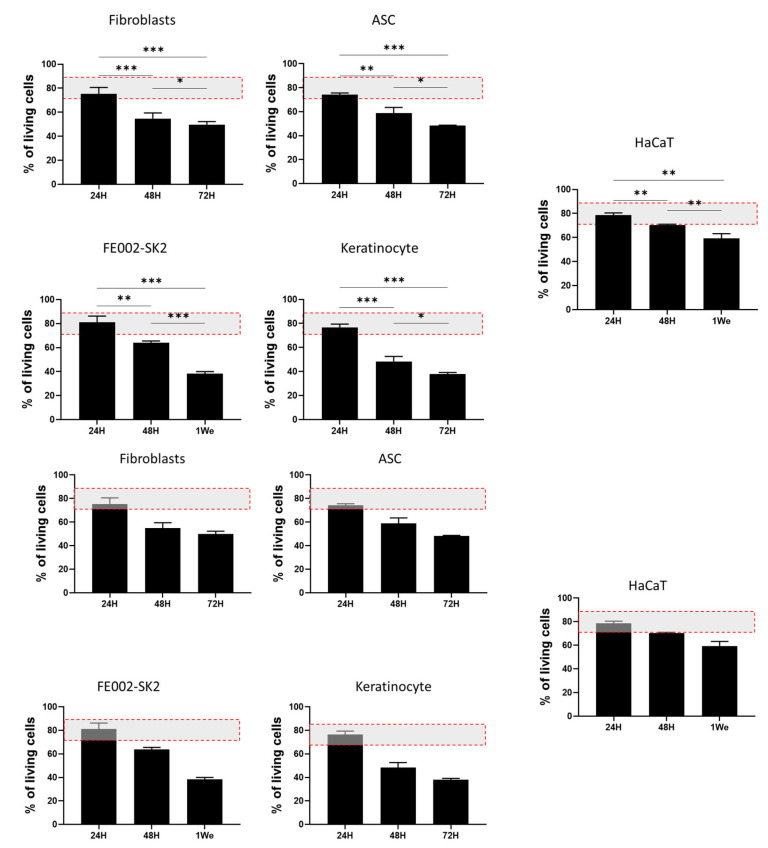
Live/dead evaluation of cellular survival in RD1 gel. A total of 100,000 cells were mixed with 100 µL of RD1, deposited in 96-well plates, and kept at 37 °C in a cell culture incubator. Live/dead staining was performed after 24, 48, 72 h or 1 week. Cell counting of live (green) and dead cells (red) was performed with the ImageJ (v1.52t) program. An example of images for cellular live/dead counting can be found in the Supplementary Figure S3. Experiments were conducted in triplicate (n = 3). The red dotted rectangle illustrates the range of cellular viability usually accepted for cell therapies. Statistical comparisons between groups were performed using GraphPad Prism unpaired two-tailed *t* tests. Significant differences are indicated by * *p* < 0.05, ** *p* < 0.01, *** *p* < 0.001.

To assess the cell delivery potential of RD1 gel for burn treatment, different cell sources relevant for burn care (polydactyly fibroblasts and ASC; FE002-SK2, keratinocyte, and HaCaT) were mixed within the gel in the absence of culture medium (starving conditions) for 24, 48, 72 h, or 1 week at 37 °C, in a cell culture incubator. The absence of medium is explained by the fact that, when used in the clinic, cells need to be delivered without culture medium for safety and regulatory compliance: culture media often contain components that are not approved for direct human use, such as animal-derived serum or growth factors. Results of the live/dead evaluation shown in Figure 5 present a survival rate of almost 80% after 24 h for all the cellular sources tested. After 48 and 72 h, we observe a decrease in cellular viability of around 20% per day for polydactyly fibroblasts and ASC, as well as for primary adult keratinocytes. FE002-SK2 cells show a better survival rate after 48 h than the other primary cells (64% vs. 58% (ASC), 54% (fibroblasts), and 48% (Keratinocytes)), respectively, and at least 40% of cells were still viable after 1 week in the gel. HaCaT immortalized keratinocytes presented a higher cellular survival rate at 48 h and 1 week than the other cell types, but this is an immortalized cell line, which is used for wound healing routinely for in vitro studies.

**Figure 6:**
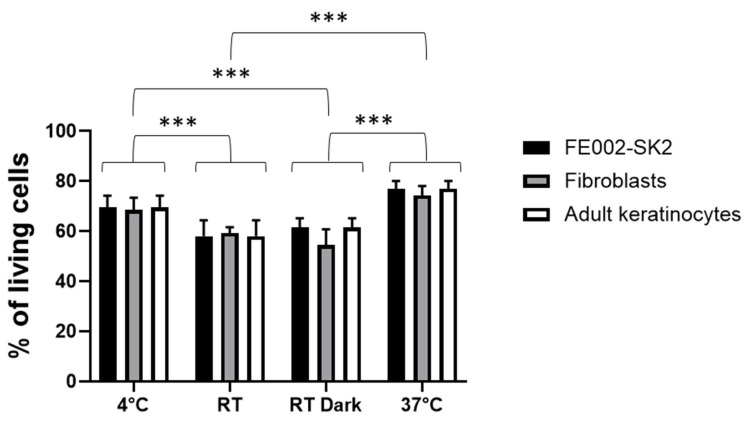
Live/dead evaluation of polydactyly fibroblasts, FE002-SK2 clinical cells, and adult keratinocyte cell survival in RD1 stored in different conditions after 24 h. A total of 100,000 cells were mixed with 100 µL of RD1, deposited in 96-well plates, and kept at 4 °C, RT (room temperature), RT in the dark, and 37 °C in a cell culture incubator. Live/dead staining was performed after 24 h. Cell counting of live (green) and dead cells (red) was performed with the ImageJ program. An example of images for cellular live/dead counting can be found in the Supplementary Figure S3. A two-way analysis of variance (ANOVA) was conducted with GraphPad Prism to examine the effects of conservation conditions on the different cell lines; significant differences are indicated by *** *p* < 0.001. Experiments were conducted in triplicate (n = 3).

As all cells show a survival of almost 80% at 37 °C, we aimed to test different storage conditions: 4 °C, room temperature (RT), RT in the dark, and 37 °C in the cell culture incubator for three different cell sources, corresponding to the ones used in the clinic to treat burn patients. Figure 6 highlights that the best storage conditions are at 4 °C or 37 °C for all the tested cell sources. A two-way analysis of variance (ANOVA) was conducted to examine the effects of conservation conditions (4 °C, room temperature (RT), RT in the dark, and 37 °C) with GraphPad Prism. The analysis showed significant main effects for conservation (*p* < 0.0001). Post-hoc Tukey test showed that the storage conditions 4 °C and 37 °C were significantly superior to the other two (RT and RT in the dark). This indicates that in the case of a delay in cell delivery in the operating room, cellular preparation could be stored for 24 h at 4 °C until used.

### 3.6. Evaluation of Cell Delivery on a De-Epidermialized Dermis (DED) for Cutaneous Cell Therapies

**Figure 7:**
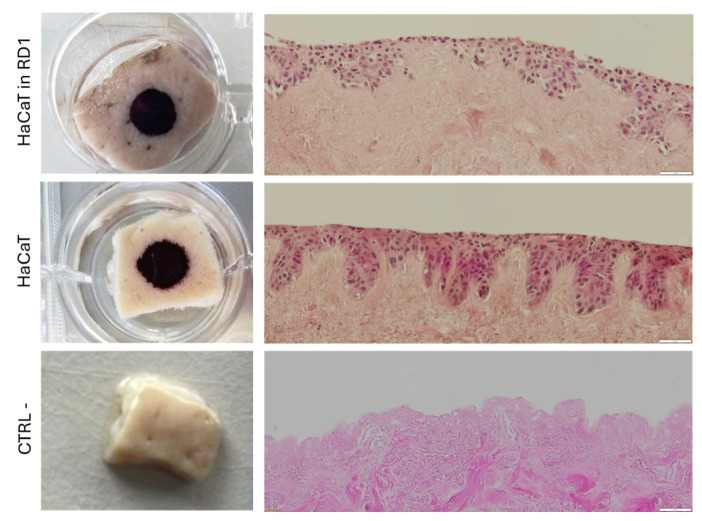
Evaluation of HaCaT cellular delivery on a DED model. 1.5 cm^2^ DED were taken from stored batches at 4 °C and deposited reticular dermis facing down onto a perforated metal support. A glass insert was carefully positioned in the center of the DED; cells suspended in medium or RD1 were added to the insert. DEDs were incubated for 3 days, then the inserts were removed. The constructs were maintained for 1 additional week at 37 °C and 5% CO_2_. DED samples were stained with MTT to assess cellular viability and homogeneity. The MTT-stained DED samples were then fixed in formalin for histological analysis and subsequent hematoxylin and eosin (H&E). (**left**) Macroscopic images of MTT staining of DED samples, (**right**) H&E staining of cross-sections of the DED samples. A total of 100,000 HaCaT cells were mixed with 100 µL of RD1, and 50 µL of the mixture was deposited on the bottom of the DED. A total of 50,000 HaCaT cells in medium were deposited on a glass insert on the bottom of the DED; CTRL-Negative control DED with no cells. Scale bar = 50 µm.

We first evaluated the possibility of delivering HaCaT keratinocyte cells on an air-liquid interface DED model [45]. MTT staining of the DED presented no observable difference between viable HaCaT cells mixed with the RD1 gel pattern distributions and the HaCaT cells in a medium that was used as the control (Figure 7). This was confirmed by the H&E staining of DED cross-sections, which showed the same cellular distribution.

**Figure 8:**
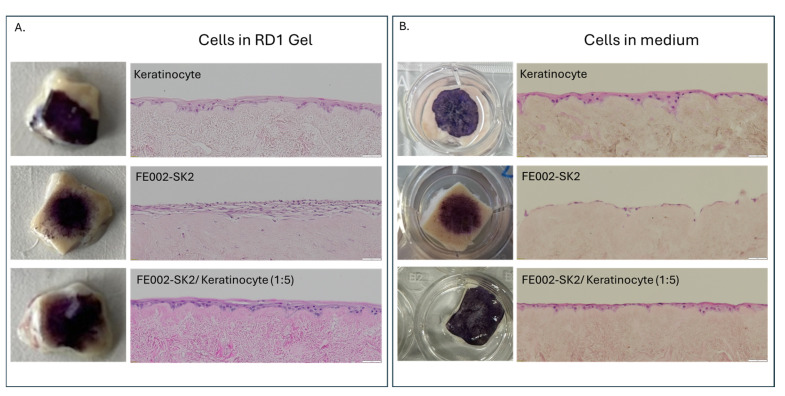
Evaluation of cell delivery on a DED model. Progenitor primary fibroblasts (FE002-SK2) and/or primary keratinocyte cells were deposited on top of a decellularized skin. (**A**) Macroscopic images of MTT staining of DED samples with cells mixed with RD1 without medium, and the H&E staining of cross-sections of the DED samples. (**B**) Macroscopic images of MTT staining of DED samples with cells seeded on top of the DED in medium, and the H&E staining of cross-sections of the DED samples. Scale bar = 50 µm.

Using the DED model, we then tested the delivery of cells that could be used in the clinic for the treatment of severe burns, such as adult primary keratinocytes and progenitor FE002-SK2 fibroblast cells. We observed that cells mixed with RD1 gel showed the same skin equivalent appearance as cells directly seeded on the DED, confirming the possibility of delivering cells with RD1 (Figure 8). Moreover, cells delivered in RD1 presented more cellular layers than those directly seeded in culture medium.

## 4. Discussion

This study investigated the feasibility of using a commercially available hyaluronic acid (HA) gel as a delivery vehicle for cells in the context of burn treatment. We employed RD1 gel, a CE-MDR-approved, class III injectable medical device formulated with 1.5% *w*/*w* of high-quality, high molecular weight, non-animal origin hyaluronic acid and 0.3% *w*/*w* lidocaine. This gel is routinely used for intradermal injections.

First, we examined the effects of RD1 at clinically relevant doses (100 μL, equivalent to a bolus) on primary cells in culture. Our results demonstrate that this HA hydrogel significantly promotes the proliferation of juvenile fibroblasts and adipose-derived stem cells (ASC) over time. Moreover, the same dose enhances collagen synthesis in juvenile fibroblasts and FE002-SK2 progenitor cells obtained from a clinically validated cell bank used for burn patient treatment [47]. The stimulatory effect of HA on collagen production has been previously documented in vivo following dermal filler injections [52], in animal models [53], and in skin organ culture systems [54], but not in isolated cell cultures. Importantly, RD1 did not induce collagen synthesis in juvenile ASC nor did their differentiation into adipocytes. This represents a safety advantage for burn treatment, particularly in third-degree burns where the gel may contact adipose tissue, as adipogenic differentiation would be undesirable. Previous studies have reported that a cross-linked HA gel with a higher HA concentration (25 mg/mL), combined with adipogenic differentiation medium, promotes ASC differentiation into adipocytes [55]. However, given the substantial differences in experimental conditions, direct comparison is not appropriate.

**Figure 9:**
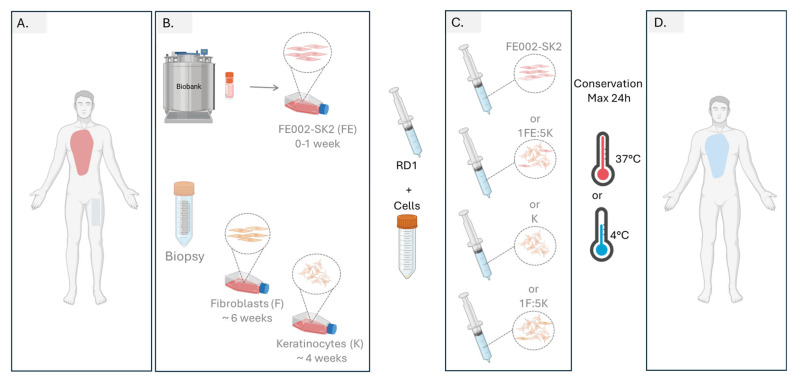
Proposed workflow for burn management with cells delivered in RD1 gel. (**A**) Burned patient arrival, after burn evaluation, a biopsy is taken for autologous fibroblast and keratinocyte cell culture. (**B**) Progenitor FE002-SK2 cells are thawed, and cells can either be used directly or, if more cells are needed for large burn surfaces, a maximum of 1 week is required to reach the desired cellular amount for treatment. Cellular amplification of autologous fibroblasts and keratinocytes needs around 4 and 6 weeks, respectively, to reach enough cells for patient treatment. (**C**) On the day of the treatment, either cellular combination can be prepared by mixing cultured cells with RD1 gel and transferring cells to the operating room. (**D**) Gel with cells should be applied during the first 24 h after preparation.

Next, we assessed the potential of RD1 as a cell delivery system. Previously, we demonstrated the feasibility of delivering primary progenitor tenocytes using HA gels employed for clinical viscosupplementation [56]. Similarly, cells mixed with RD1 exhibited high viability (~80%) after 24 h of storage at either 37 °C or 4 °C. This suggests that, in a clinical setting, the cell–gel preparation could be stored in an operating room refrigerator until patient readiness. We used a decellularized skin model for testing cellular delivery of different primary cellular combinations mixed in RD1 gel and compared them to cells directly seeded on the top of the DED. We have demonstrated that RD1 could be an alternative to the collagen matrix that is currently used in the clinic for the delivery of FE002-SK2 progenitor fibroblasts [47,57], for early burn coverage. We recently demonstrated that a combination of one fibroblast (progenitor or adult) and five adult keratinocytes delivered in culture medium on a DED model resulted in multilayered keratinocyte structures [45]. In this study, we show that the same 1F:5K ratio delivered in RD1 gel achieves comparable or superior outcomes, supporting the concept that HA-based delivery could be a viable option for burn patient treatment. A conceptual clinical pathway highlighting the potential use of RD1 as a delivery vehicle for therapeutic skin cells in burn management is presented in Figure 9, illustrating its translational relevance for regenerative medicine applications.

There are several products available that are hyaluronan-based for the topical management of burn wounds. Cell-free products include Hyalomatrix^®^, Hyalosafe^®^, HYAFF^®^-11, and Ialugen^®,^ while cell-laden products such as Hyalograft 3D™ and Laserskin™ are also available [36,58,59,60,61], and several clinical studies have shown their benefits for their use on burn wounds [62,63,64,65,66]. Hyaluronan hydrogels are easy to apply to the wound and provide a smoothing effect, which may contribute to the significant alleviation of pain noticed by many patients [67]. Still, hyaluronan hydrogels may have moderate outcomes depending on the extent and severity of burn injury. Topical delivery of HA has shown variable tissue penetration capacities, wherein a MW of 100 kDa enabled optimal passage through the disrupted skin barrier [68,69]. Hence, if large surfaces of “intact” skin remain throughout the burn injury, there may be variable clinical results. This was shown in a recent study by De Francesco in a burn patient with a formulation of 0.2% HA needing more than 4 weeks for partial wound closure [37].

Hyaluronan viscous solutions such as RD1 can also be used to incorporate therapeutic cells, forming a 3D environment with enhanced biological activities and wound healing potential [70,71,72]. The 3D cell incorporation could also potentially reduce the inherent immunogenicity of the scaffold formulations [73]. Furthermore, the use of RD1 may offer several practical advantages. The ease of manufacturing and sterile delivery of these hydrogels could streamline clinical application. Additionally, the potential for customization, such as incorporating specific growth factors or antimicrobial agents, could allow for personalized treatment approaches tailored to individual patient needs. Similarly, autologous patient cells or off-the-shelf cells within highly controlled cell banks could be proposed.

## 5. Conclusions

Taken together, these findings and our past data support the use of non-crosslinked hyaluronan RD1 as a versatile and effective vehicle for cell delivery. RD1 not only protects the cells but also provides the ideal viscosity to remain at the injured burn site, which is not the case with cells delivered in NaCl. Notably, the HA solution also showed stimulation of cell growth and collagen synthesis. Its ease of use, compatibility with various cell types, and ability to support therapeutic outcomes make it a strong candidate for applications in wound healing, burn management, and regenerative medicine. Future research should explore its performance in long-term in vivo models, its interaction with bioactive molecules, and its scalability for clinical translation. In addition, experiments employing larger gel volumes (5–10 mL) are needed to enable coverage of extensive burn areas and to model real-life conditions. Finally, clarification of the regulatory framework for this “combination product” is required to ensure appropriate pathways for patient use.

## Data Availability

The original contributions presented in this study are included in the article/supplementary Materials. Further inquiries can be directed to the corresponding authors.

## Supplementary Materials

The following supporting information can be downloaded at: https://www.mdpi.com/article/10.3390/pharmaceutics18010021/s1.

## Abbreviations

DED	de- epidermialized dermis
DMEM	Dulbecco’s modified Eagle medium
DMSO	dimethyl sulfoxide
CSI	cell strainer inserts
HA	hyaluronic acid
ASC	adipose stem cells
RD1	redensity 1 hydrogel
ECM	extracellular matrix
FDA	food and drug administration
