# Supplementary material for: Non-Crosslinked Hyaluronic Acid Redensity 1® Supports Cell Viability, Proliferation, and Collagen Deposition in Early Burn Management
Source: Pharmaceutics. 2025 Dec 23;18(1):21. doi: 10.3390/pharmaceutics18010021 (PMC12845186; doi:10.3390/pharmaceutics18010021)
Supplement: Supplementary file 1 [file pharmaceutics-18-00021-s001.zip › pharmaceutics-4027673-supplementary.pdf]

pharmaceutics

Supplementary Material

Non-Crosslinked Hyaluronic Acid Redensity 1® Supports Cell Viability, Proliferation, and Collagen Deposition in Early Burn Management

Zhifeng Liao[1,2], Xi Chen[1,2], Romain Brusini[3], Jimmy Faivre[3], Lee Ann Applegate[1,4,5], Killian Flegeau[3*†] and Nathalie Hirt-Burri[1,2*†]

Supplementary figures

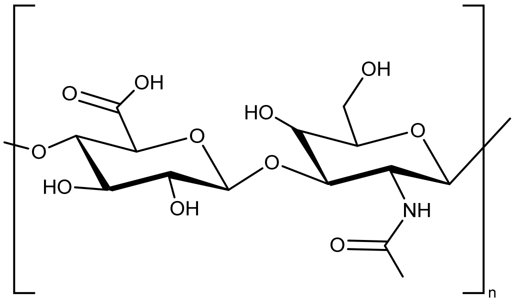

Figure S1: Chemical structure of hyaluronic acid (HA). HA is a high–molecular weight, non-sulfated glycosaminoglycan composed of repeating disaccharide units of D-glucuronic acid and N-acetyl-D-glucosamine. HA is a major component of the extracellular matrix and contributes to tissue hydration, elasticity, and viscoelasticity.

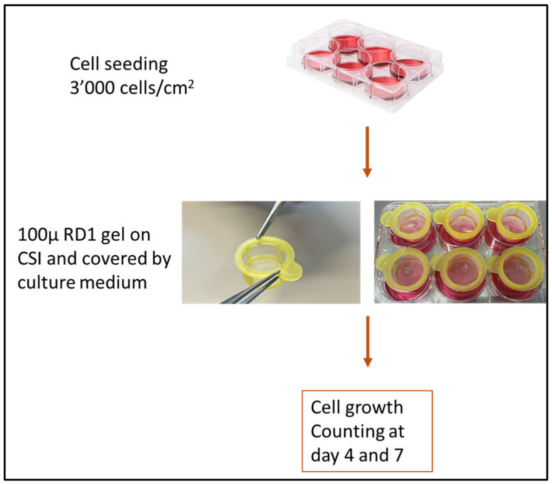

Figure S2: Experimental set up to analyze the effect of a clinical dose of RD1 gel on cells using the insert model to separate gel from direct contact with cells. Cells are seeded into 6 well plates at 3,000 cells/cm2. RD1 gel was overlayed into the insert and covered with cell culture complete media. Cell growth was obtained by enzymatically recovering the cells and counting with a hemacytometer. Experiments were accomplished in triplicate.

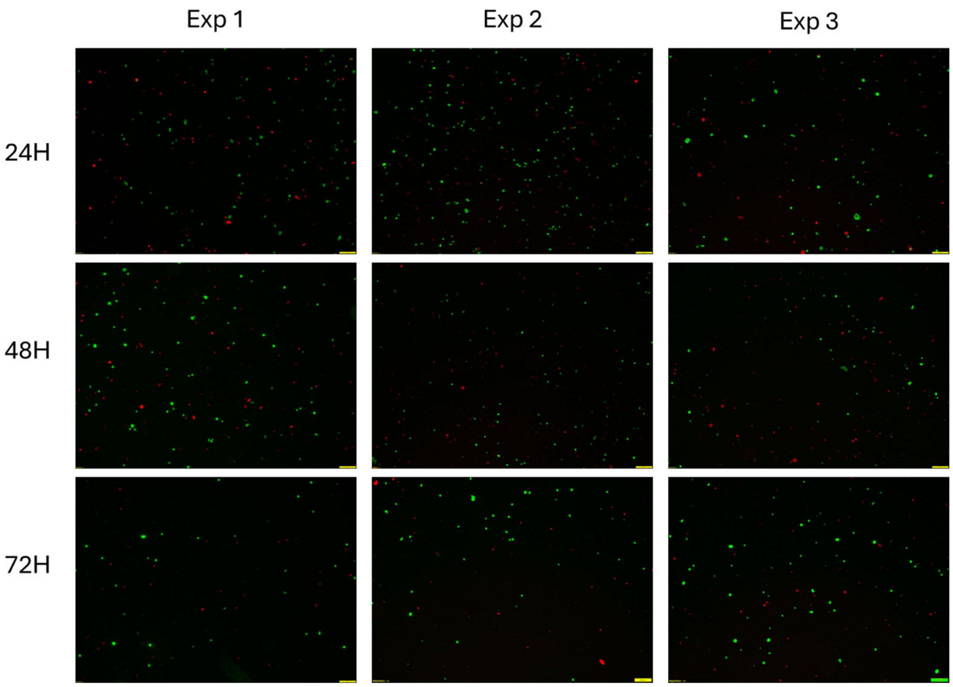

Figure S3: Example of images for cellular Live/Dead counting of 3 experiments done with polydactyly fibroblasts at 24, 48 and 72 hr of growth. Pictures were taken with a fluorescence microscope (Olympus IX83 microscope With a DP75 camera) using the appropriate filters, GFP: green fluorescence for live cells and TRITC, red fluorescence for dead cells. Cell counting (live and dead cells) was done with the Image J program. Three experiments were done for each cell source.

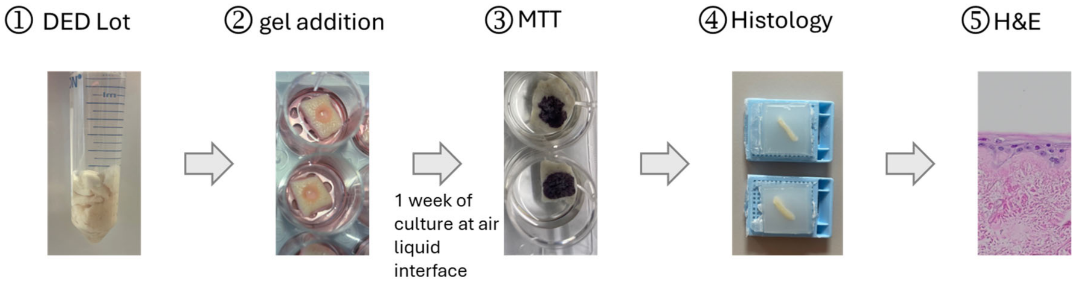

Figure S4: DED experimental setup. ① DED 1.5 cm^2 were taken from stored batches at 4°C, washed several times with DMEM and incubated for at least 1 hour in Medium 1. ② DED samples were deposited (reticular dermis facing down) onto a perforated metal support measuring 1 × 1 × 0.5 cm that were placed at the bottom of a 12-well plate. Medium was added just to let the top of the dermis uncovered to create an air liquid interface. A glass insert was carefully positioned in the center of the DED with the help of a sterile forceps for the cells suspended in medium, then cells were added on the insert or directly on the DED. The plates with DEDs were incubated for 3 days at 37 °C and 5% CO2. Following this period, the inserts were removed, the DED culture medium was refreshed, and the constructs were maintained for 1 week at 37 °C and 5% CO_2 . ③ DED samples were stained with MTT to assess cellular viability and homogeneity ④The stained DED samples were then fixed in formalin for histological analysis and sub-sequent hematoxylin & eosin (H&E) staining.

https://doi.org/10.3390/pharmaceutics18010021

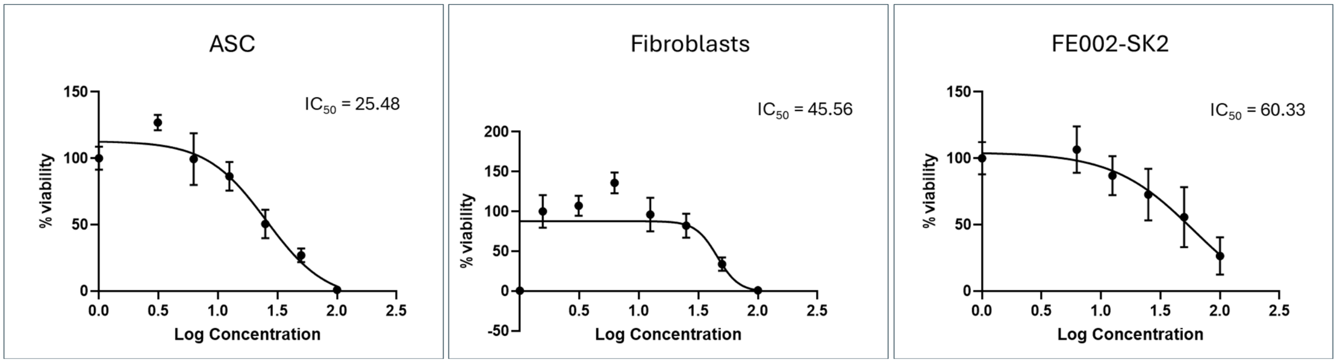

Figure S5: IC_{50} calculation: Dose–response curves were generated by plotting compound concentration against percent inhibition. Nonlinear regression using a four-parameter logistic model was performed in GraphPad Prism to fit the data The half-maximal inhibitory concentration (IC_{50}) was calculated from the fitted curve and is indicated on the graph

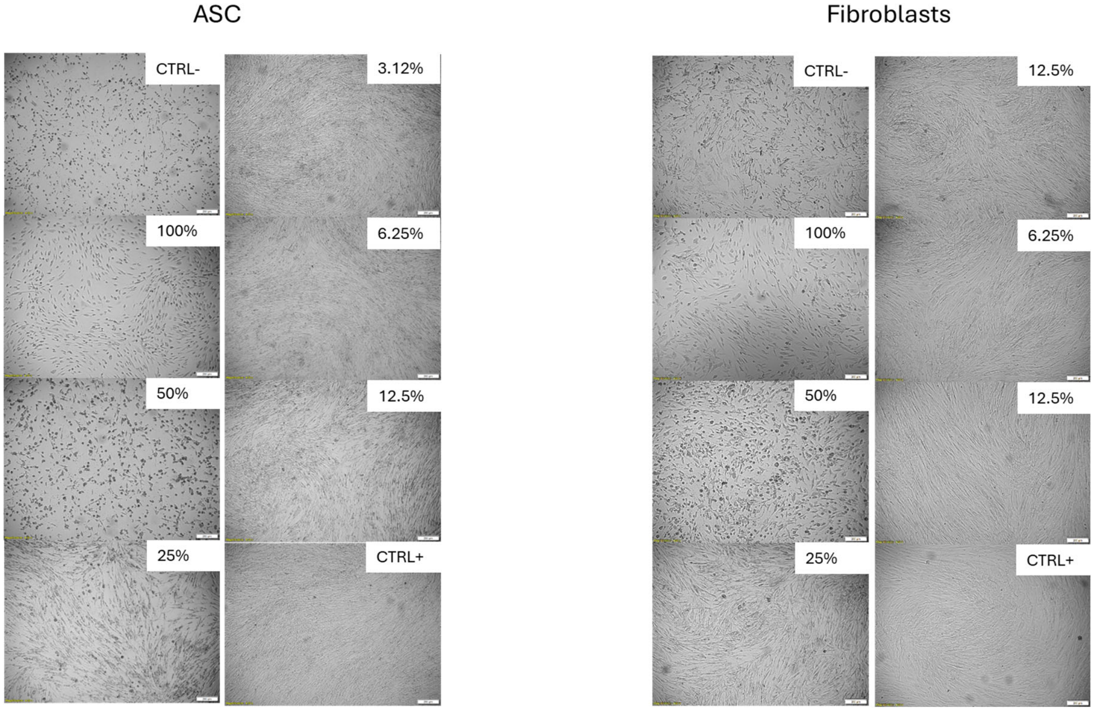

Figure S6: Images of polydactyly fibroblast and ASC at the end of the cytotoxicity assay, before the cellTitter assay. The RD1 gel concentrations are indicated in percents on the top left of the pictures. Polydactyly primary ASCs and fibroblasts cultured in their respective growth medium are indicated as positive control (CTRL+) and cells in PBS are the negative control (CTRL-). Scale Bar = 200 µm

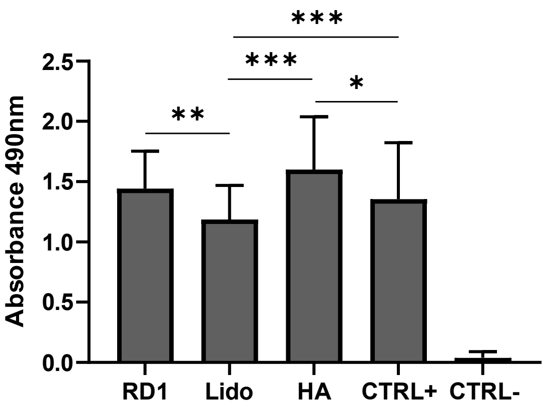

Figure S7: CellTiter result of polydactyly primary fibroblasts treated with 20% RD1, 20% 0.3% lidocaine, 20% HA for 24 hours. RD1 gel (with lidocaine) shows no statistical difference than with HA alone or with the positive control.

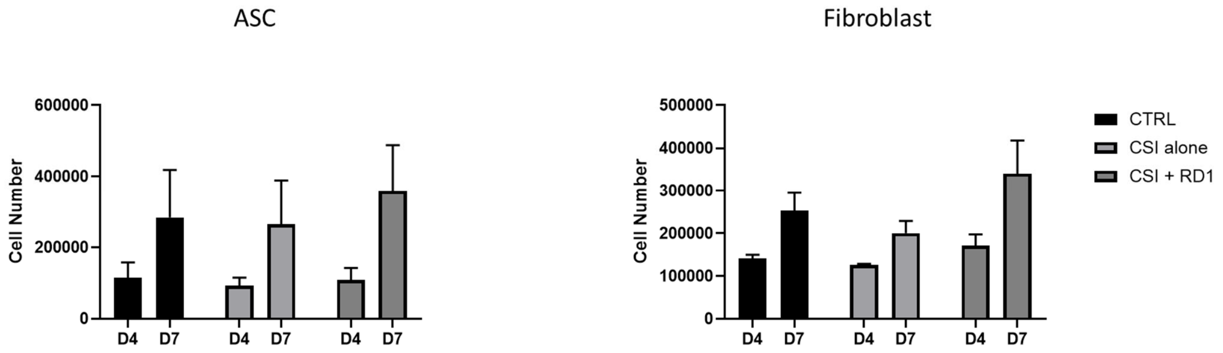

Figure S8: Cellular growth evaluation by cellular enumeration at day 4 and day 7 of polydactyly primary ASCs and fibroblasts cultured in their respective growth medium with the presence of a CSI, with or without 100 µL RD1 gel.

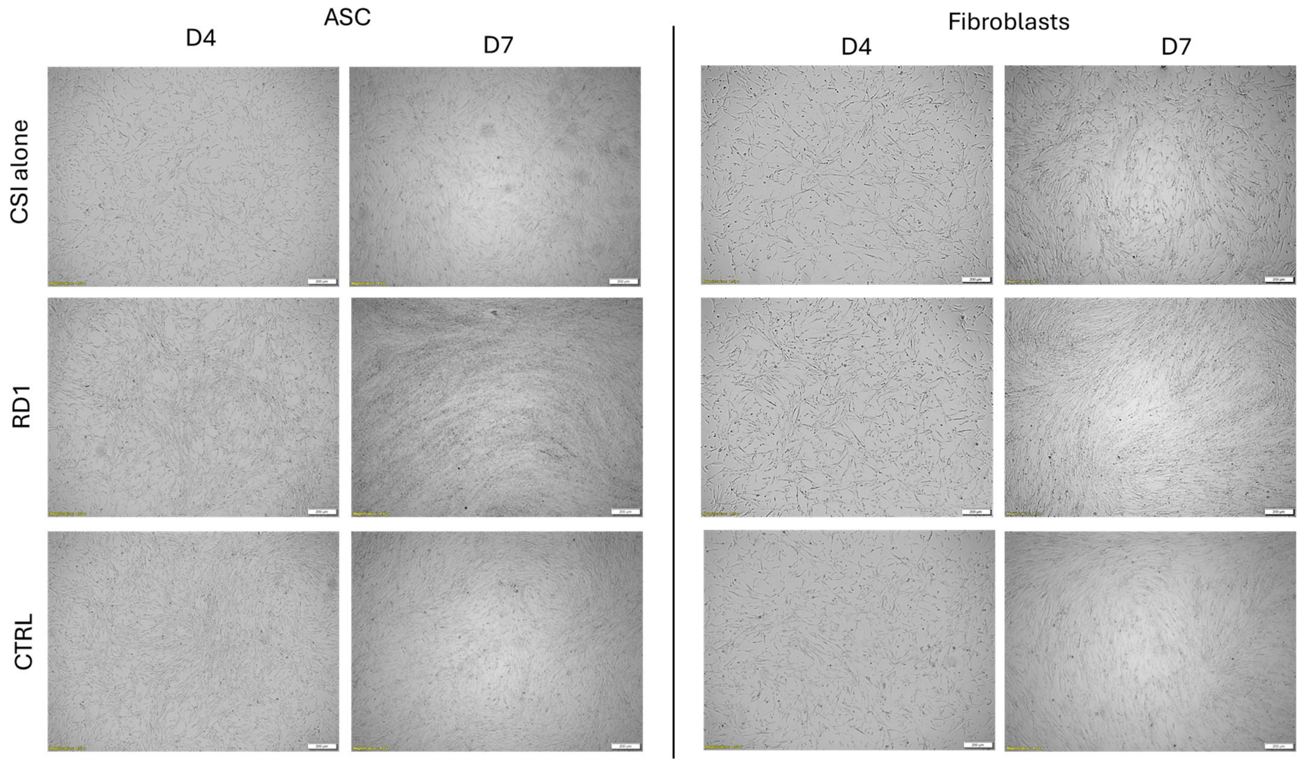

Figure S9: Pictures of cells (Primary polydactyly Adipose Stem Cells and Fibroblasts) in culture before cellular enumeration for cell growth evaluation at Day 4 and 7. Cells with the insert only (top), with the insert and the RD1 gel (middle) and cells with only cell culture media (bottom) Scale bar =200µm

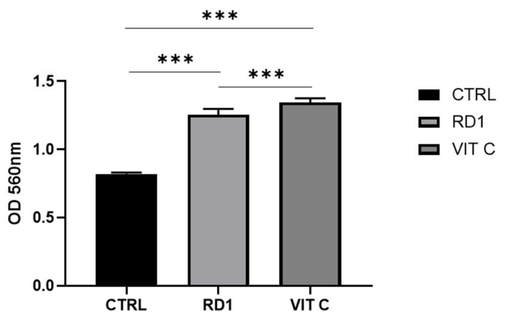

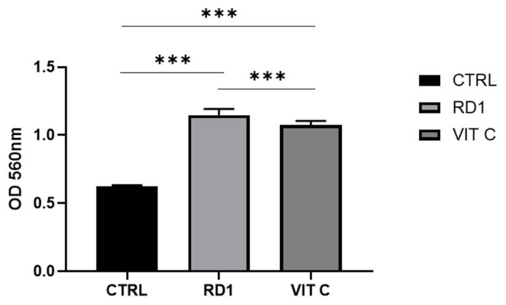

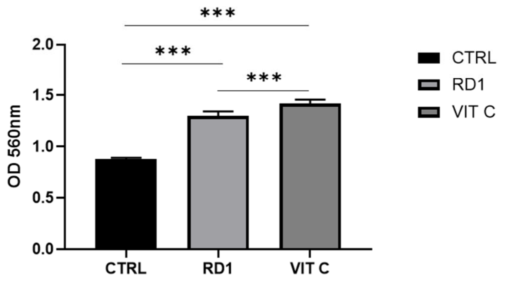

https://doi.org/10.3390/pharmaceutics18010021

Figure S10: Collagen quantification by Sirius Red optical density measurements of primary polydactyly fibroblasts. Raw OD_{560} visible absorbance (which corresponds to collagen content) was measured for three independent experiments.

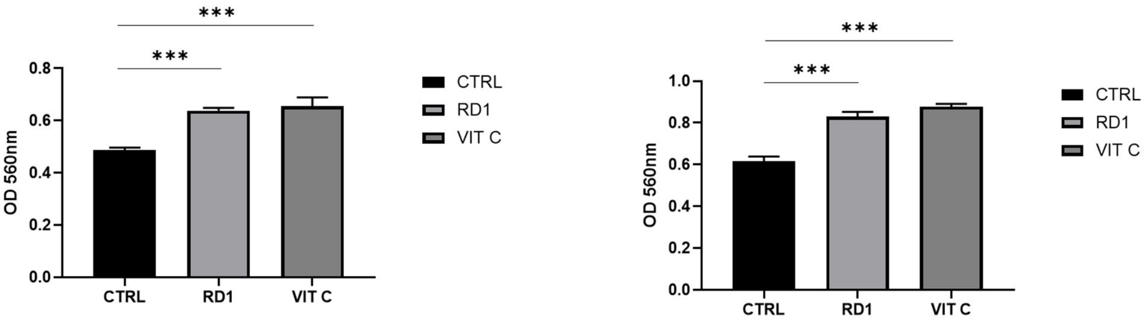

Figure S11: Collagen quantification by Sirius Red optical density measurements of FE002-SK2. Raw OD_{560} visible absorbance (which corresponds to collagen content) was measured for two independent experiments.

https://doi.org/10.3390/pharmaceutics18010021
